# Policies and socioenvironmental dynamics for ecosystem management

**DOI:** 10.1016/j.mex.2023.102205

**Published:** 2023-04-28

**Authors:** Vargas del Río David, Brenner Ludger

**Affiliations:** a Department of Habitat and Human Development. Periférico Sur Manuel Gómez Morín 8585, Instituto Tecnológico y de Estudios Superiores de Occidente, Santa María Tequepexpan, San Pedro Tlaquepaque, Jal 45604, Mexico; b Unidad Iztapalapa, Department of Sociology, Universidad Autónoma Metropolitana, Avenida San Rafael Atlixco 186, Mexico City 09340, Mexico

**Keywords:** Conservation, Socioenvironmental evaluation, Development, Governance, Community-based lands, Historical political ecology, Local production-based management, Method for relating domestic policies and socioenvironmental dynamics

## Abstract

The continuous loss of biodiversity has extended the Convention on Biological Diversity's target towards safeguarding 30% of the planet by 2030 with some form of protected area management. This is a challenge, considering the poor compliance of the Aichi Biodiversity Targets reported in several assessments, and that 37% of the remaining unprotected natural areas are inhabited by indigenous and local communities. Modern conservation policies tend to convert areas destined for protection into complex socioecological landscapes, so it is critical to develop policies capable of establishing long-term harmonious relations between local societies and their environments. Despite the fundamental importance of defining this interrelation, methodologies for evaluating it are still unclear. We propose a method for assessing the outcome of policies in socioenvironmental practices based on a historical-political ecology analysis of a region, the construction of socioenvironmental scenarios, and comparing populations scattered through the study area. Each “scenario” is a relation between nature and society after a shift in public policies. Conservation scientists, environmental managers, and policymakers can use this methodology to assess old policies, design new ones, or map the socioenvironmental dynamics in their area of interest. Here, we detail this approach and illustrate its application in the coastal wetlands of Mexico. The method can be outlined as follows:

• Deduce socioenvironmental epochs for a region by analysing its historical political ecology.
• Analyse the socioenvironmental dynamics in selected case studies scattered through the region.
• Use the resulting scenarios as conceptual bridges between internal policies and current socioenvironmental dynamics.

## Graphical abstract

**Figure:**
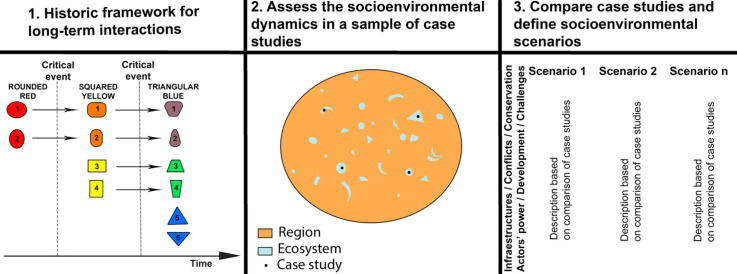

Specifications table

**Table:** 

Subject area:	Environmental Science

More specific subject area:	Environmental Management
Name of your method:	Method for relating domestic policies and socioenvironmental dynamics
Name and reference of original method:	Historical Political Ecology
Resource availability:	Data will be made available on request

## Details of the method

Biodiversity conservation is a worldwide priority that requires massive investments to efficiently protect 30% of the Earth's surface by 2030 [1]. Actions in this area are the principal response to the challenges that arise from inadequate relations between society and nature, but more than money and decrees are needed. Proof of this is the poor compliance with biodiversity goals reported in the Aichi Biodiversity Target (see [[2], [3], [4]]). Current conservation policies are based on environmental restrictions as an increasing number of actors seek to conciliate (successfully or not) their often-conflicting interests under the concept of good governance. But this strategy often creates politized environments (see [[5], [6], [7], [8]]; and [9], for additional details). Thus, it is crucial to understand the socioenvironmental dynamics that tend to contribute to conservation and those that do not, and to evaluate the long-term consequences of environmental policies [10]. Sadly, well-defined, applicable methodologies capable of deepening our understanding of the relation between government policies and socioenvironmental dynamics are still lacking [11,12].

Environmental management is critical for nature conservation and engaging methodologies based on qualitative and quantitative scientific traditions [13]. Qualitative studies can effectively describe local visions, socioeconomic activities, and relations between local users and environmental conservation (e.g., [[14], [15], [16]]), but often lack rigorous, systematic comparisons with other case studies. This impedes detecting common patterns and may hinder the construction of adequate theories and the implementation of public policies. As a result, recommendations generally fail to transcend local contexts [11], and rarely go beyond normative and widely-accepted, but ambiguous and controversial, concepts, such as “social participation” or “good governance” [12].

Quantitative studies are usually carried out on a broader geographic scale [11], generally based on indicators related to biodiversity and environmental services associated with distinct patterns of socioeconomic development (e.g., [[17], [18], [19]]). However, their contribution to day-to-day resource management is limited, due to a lack of specificity and difficulty in quantifying people's perceptions and subjectivities [11]. Assessments of this kind are often unsatisfactory when it comes to explaining specific socioenvironmental processes that trigger environmental degradation. In short, quantitative studies tend to highlight the importance of restrictive protection measures, without considering power struggles, agrarian conflicts, and local-level resistance.

The gap between these scientific traditions impedes achieving a deeper understanding of complex relations between public policies and local socioenvironmental dynamics. Without this knowledge, governments may create protected areas in fragile environments that prove unsuccessful, modify local institutions, and spark severe social conflicts [5]. They may also restrict agricultural use, livestock-raising, hunting, or logging for self-consumption, without considering other, more harmful activities, such as land privatization and sales [20]. For these reasons, we propose a method that systematically bridges this gap to assess existing public policies and design new ones. Our method also makes it possible to describe socioenvironmental dynamics that affect resource use and management.

**Fig. 1:**
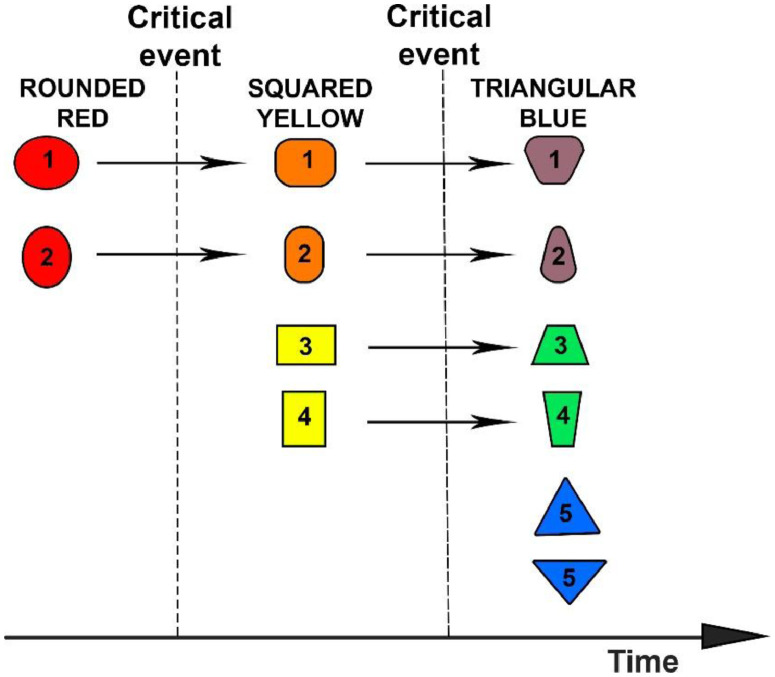
Illustration of the transformation of the relation between society and nature over time.

Our proposal is based on historical-political ecology, which holds that social conflicts and public demands modify practices involving nature and may trigger critical political reforms (e.g., [[21], [22], [23], [24], [25]]). The societies that emerge after such alterations in decision-making and power relations establish a new structural relation with nature and endure in this state for many years until a new political transformation occurs ([25], see also [26]). One outcome of this understanding of the relation between society and nature is that the inhabitants of one political entity, in the same epoch, and in analogous natural environments, will share related practices regarding nature (see Fig. 1). If we suppose that red ellipses numbers 1 and 2 represent communities established in a certain region and epoch when political agreements promote red-rounded forms of socioenvironmental relations, then they will persist under this form a subsequent critical event changes this political context and the region enters a new phase of socioenvironmental relations, represented here by the square-yellow forms. As a result of this political transformation, communities 1 and 2 will adapt their social structure and transform into societies with orange-rounded/square forms of socioenvironmental relations, manifesting behaviours distinct from those of communities 3 and 4, which exist in the same region as communities 1 and 2 but were forged in this new epoch. Posterior to this first transformation, a new critical event at the regional level may modify the socioenvironmental practices and foment triangular-blue forms of socioenvironmental relations. The new communities 5 and 6 will be blue triangles, while communities 1 and 2 will become brown, rounded trapezoids, and communities 3 and 4 will adapt their behaviour as green trapezoids.

This understanding of socioenvironmental transformations makes it possible to establish a relation between policy changes and the associated socioenvironmental behaviour of communities in a given ecosystem. Researchers should first identify critical moments of social instability in the political entity by analysing historical sources on changes in land-use policies and their consequences. They can then characterize the socioenvironmental dynamics of a group of populations in that entity, including their transformations after each critical event. Finally, they must systematize this information and generalize their findings to all the communities in the political entity. The result is a link between domestic policies in the entity and the local socioenvironmental dynamics they produce.

To identify critical transitions in geographical entities, it is necessary to analyse their histories in search of crucial changes in public policies. These moments tend to manifest themselves in intense social conflicts that precede critical turning points. Typical indicators are marked changes in property rights, disputes over access to, and the use of, natural resources, or substantial investments in infrastructure. In developing countries, transformations of this kind involve support for the commercial exploitation of nature (agriculture, fishing, mining, protected areas, tourism, or industrialization, amongst others), often triggered by state actors supported by multilateral organizations, businesses, or NGOs [27]. Changes might also involve critical modifications in legislation. Consequences may include revolutions, coups, structural economic reforms, and migrations.

Once those turning points are identified, it is important to develop a general framework to analyse long-term interactions between (changing) public policies and socioenvironmental dynamics, along with the political ideologies that each period seeks to implement on the ground (Table 1). In other words, we recommend that researchers identify the ideals, principles, and doctrines that define and shape public policies, and consider wide-ranging consequences for environmental management.

**Table 1:** Elements for constructing a framework for long-term interactions between public policies and socioenvironmental dynamics.

Critical event 1	Policies	Political ideology	Political ecology
Critical event 2	Policies	Political ideology	Political ecology
Critical event 3	Policies	Political ideology	Political ecology
Critical event n	Policies	Political ideology	Political ecology

Vargas del Río's [28] study of conservation policies in Mexico, for example, described the historical political ecology of the country by tracing three critical events (see also the Research Article):

(1) Implementation of the Constitution in rural areas after the Revolution (1917 onwards), under a communist approach. The policies derived from this legal basis for organizing the country produced a land reform program that established agrarian communities on a massive scale and granted them control over extensive territories under community-based management strategies. These *ejidos* and *comunidades indígenas* (in Spanish) had only a limited capacity to forge links with businesses or industries, so they developed mostly subsistence-level extractive practices and economies based on use value. This political ecology is fundamental for conservation in Mexico because it extends over 50% of the land surface, where 80% of well-conserved forests exist.
(2) Post-war industrialization was promoted after 1945 under a statist approach. Financed by International banks, the Mexican State invested in highways, airports, electricity, extractive industries, and tourism centres in coastal areas, while slowing land reform and amending some laws that restricted the access of businesses and industries to communal territories. Rural populations received industrial techniques, products, economic incentives, education, and training. As a result, extractive practices intensified, especially around the industrial centres.
(3) The structural neoliberal reforms implemented from 1982 onwards entailed significant political and economic changes, brought large-scale importation of raw materials, legalized the privatization and sale of community-owned lands, and relaxed the restrictions previously imposed on businesses. New actors engaged in nature conservation imposed environmental regulations and instrumented new modes of environmental management and protection. The result was an economic crisis that affected many communal territories, and triggered land sales, tourism development, extractive activities, and the establishment of numerous protected areas.

**Fig. 2:**
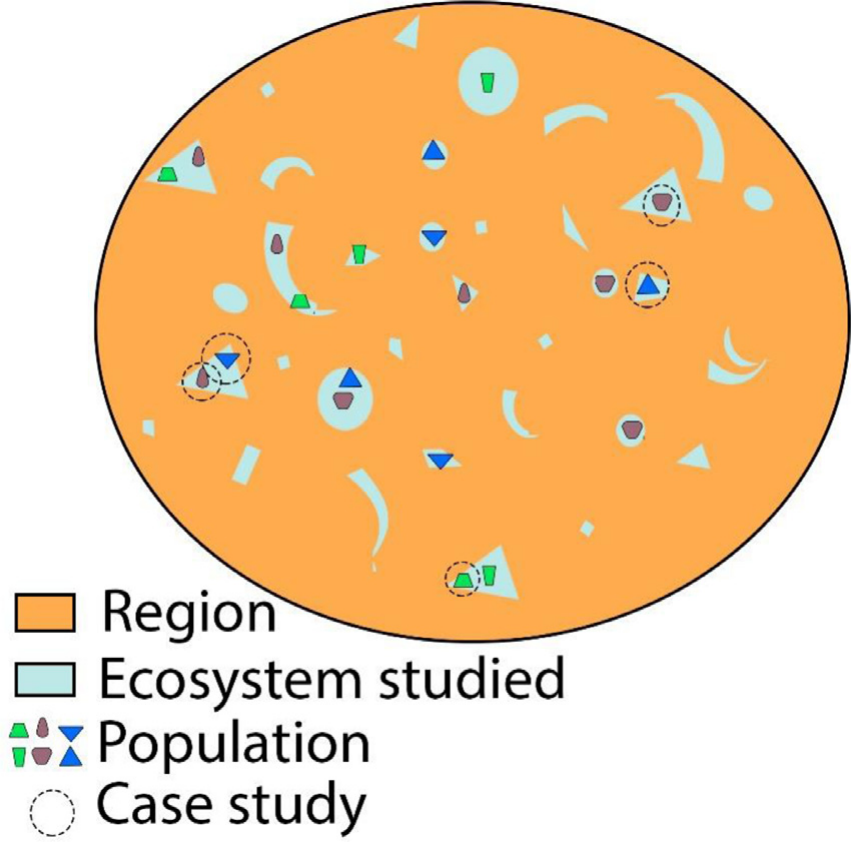
The selection process of case studies in the ecosystem studied based on historical and geographical criteria.

After constructing a framework of this kind, researchers should select case studies (localities) in the ecosystem chosen, applying two selection criteria: one based on a historical conceptualization that includes the case studies found in all the epochs previously deduced from the historical analysis; the other on choosing case studies distributed within the political region by applying an adequate sampling criterion; options include quota, convenience, purposeful, geostatistical, or random. Fig. 2 uses the representations from Fig. 1 to illustrate the selection process. As can be seen, this researcher, aided by the historical framework (Fig. 1) selected six case studies –two per epoch (with similar colour and form)– in the ecosystem under study (green areas), from different zones in the region. Vargas and Brenner [20] selected eight case studies, recovered from two independent research projects conducted previously in mangroves in Mexico [29,30]. Those studies were carried out using similar methodologies and were distributed in four of the five mangrove regions marked in the biogeographical regionalization [31]; namely, Gulf of Mexico, Yucatan Peninsula, Central Pacific, and Southern Pacific (see Fig. 1 in [20]).

Once the studies are selected, researchers should assess the socioenvironmental dynamics in each one. Key topics include productive activities in the ecosystem, social organization, the actors involved, and economic demands. We consider that holding semi-structured interviews with all relevant resource users is the most convenient research method. Asking elderly people questions about past events may be an adequate approach in localities that have gone through multiple historical periods. As an example, in their study of socioenvironmental dynamics in mangrove areas, Vargas and Brenner [20], conducted 180 in-depth, semi-structured interviews with residents, representatives of local groups, NGOs, multilateral organisms, government agencies in charge of environmental conservation, and academics. They selected informants using the snowball technique.

Researchers must then transcribe the interviews, gather all relevant information, code it, and define suitable analytical categories of socioenvironmental dynamics associated with the long-term interactions associated with public policies. Vargas and Brenner [20] used Atlas. Ti 6.0 software after recording and transcribing the interviews, then formed categories related to each historical period. Based on that classification, they identified three “socioecological scenarios” characterized by specific socioenvironmental dynamics (see Table 1 in [20]).

We suggest characterizing socioenvironmental dynamics according to the “Spatial Triad” [32], a classification of space(s) that provides an insightful exploration of organizational space [33]. We also recommend describing, first, dynamics related to territory, environment, and the means of production –what Lefevbre [32] calls the physical or “lived space”– then describing the social and political dynamics – Lefevbre's social or “perceived space”– and, finally, assessing the mental or “conceived space” (see [32] for further details). The model of socioecological scenarios constructed using this method produces a description and characterization of socioenvironmental dynamics that serves as a “conceptual bridge” between domestic policies and those dynamics in specific ecosystems. It is a generalization that can be used as a tool for successful ecosystem management, based on distinct scenarios, and considering specific physical features, such as land tenure, highways, urbanization, resorts, and protected areas (Table. 2).

**Table 2:** Case of a synthetic framework of long-term interactions between public policies and socioenvironmental dynamics in a political region.

Critical event	Policies	Political ideology	Political ideals
Mexican Revolution	Article 27 of Mexico's Constitution	Communist	Land distribution / community-based resource management
End of World War II	Constitutional amendments and federal laws on the use of natural resources. / Slowing of land reform.	Statist (Fordist) capitalism	Industrial development / extractivism
Structural reforms	Constitutional amendments and federal laws opened territories to businesses. / End of land reform.	Neoliberalism	Privatization of common lands / nature conservation / free market

## Ethics statements

Informed consent was obtained from all interviewees and participants’ data was fully anonymized.

## CRediT authorship contribution statement

**Vargas del Río David:** Data curation, Funding acquisition, Formal analysis, Validation, Conceptualization, Writing – review & editing, Writing – original draft. **Brenner Ludger:** Data curation, Funding acquisition, Formal analysis, Conceptualization, Validation, Writing – review & editing, Writing – original draft.

## Declaration of Competing Interest

The authors declare that they have no known competing financial interests or personal relations that could have appeared to influence the work reported in this paper.

## Data Availability

Data will be made available on request.
